# A test of native plant adaptation more than one century after introduction of the invasive *Carpobrotus edulis* to the NW Iberian Peninsula

**DOI:** 10.1186/s12862-021-01785-x

**Published:** 2021-04-28

**Authors:** Carlos García, Josefina G. Campoy, Rubén Retuerto

**Affiliations:** 1 CIBUS, Campus Sur, Universidade de Santiago, 15782 Santiago de Compostela, Spain; 2 Department of Functional Biology, Area of Ecology, Faculty of Biology, CRETUS Inst., Universidade de Santiago de Compostela, 15782 Santiago de Compostela, Spain

**Keywords:** *Artemisia crithmifolia*, Aizoaceae, Biomass production, Co-evolutionary changes, *Helichrysum picardii*

## Abstract

**Background:**

Although the immediate consequences of biological invasions on ecosystems and conservation have been widely studied, the long-term effects remain unclear. Invaders can either cause the extinction of native species or become integrated in the new ecosystems, thus increasing the diversity of these ecosystems and the services that they provide. The final balance of invasions will depend on how the invaders and native plants co-evolve. For a better understanding of such co-evolution, case studies that consider the changes that occur in both invasive and native species long after the introduction of the invader are especially valuable. In this work, we studied the ecological consequences of the more than one century old invasion of NW Iberia by the African plant *Carpobrotus edulis*. We conducted a common garden experiment to compare the reciprocal effects of competition between *Carpobrotus* plants from the invaded area or from the native African range and two native Iberian plant species (*Artemisia crithmifolia* and *Helichrysum picardii)* from populations exposed or unexposed to the invader.

**Results:**

Exposure of *H. picardii* populations to *C. edulis* increased their capacity to repress the growth of *Carpobrotus*. The repression specifically affected the *Carpobrotus* from the invader populations, not those from the African native area. No effects of exposition were detected in the case of *A. crithmifolia*. *C. edulis* plants from the invader populations had higher growth than plants from the species' African area of origin.

**Conclusions:**

We found that adaptive responses of natives to invaders can occur in the long term, but we only found evidence for adaptive responses in one of the two species studied. This might be explained by known differences between the two species in the structure of genetic variance and gene flow between subpopulations. The overall changes observed in the invader *Carpobrotus* are consistent with adaptation after invasion.

**Supplementary Information:**

The online version contains supplementary material available at 10.1186/s12862-021-01785-x.

## Background

The large-scale alteration of species distributions is one of the most drastic types of disturbance to the biosphere that have occurred during the Anthropocene [1, 2]. Although global biodiversity is being eroded [3, 4], it may be increasing at smaller spatial scales due to the arrival of invasive species [5, 6]. The long-term ecological consequences of these invasions are unclear. While some non-native species can outcompete native species to extinction [7–9], others may cause no serious adverse impacts (as is often the case, at least in the short-term; see [10, 11], giving native species the opportunity to co-evolve with the invaders (reviewed in Oduor et al. [12]) and even to develop new mutualisms [13]. In this way, invasive species might eventually become stably integrated in the new ecosystems [14, 15], increasing local biodiversity and reinforcing the services provided by these ecosystems or their resilience to further alteration (reviewed in Chapman et al. [16]; but see Kaiser-Bunbury et al. [17]).

Analyses of the evolutionary processes that could result in this final integration of the invasive species in the long term are relatively scarce [18], but short-term experimental findings consistent with evolutionary change in invasive species, leading to divergence in relevant adaptive traits from their source populations [19, 20], are accumulating [21, 22]. Such findings run from increases in invasive ability [23–25], to changes in interactions with other species [26, 27], and responses to abiotic factors [28, 29]. Invasive species have also been shown to induce short term evolutionary changes in native species [30–36], reviewed in Oduor et al. [12]. However, these short-term changes may be poor guides to predicting the properties of future ecosystems [37, 38], because biological invasions may alter the physical ecosystem, species composition and abundance, favouring the establishment of other invasive species [39, 40] and triggering a cascade of coevolutionary, multi-species processes [18, 41], all of which may take some considerable time [37, 42, 43]. Thus, studies of biological invasions are most informative when they consider the possible changes in both the invasive species and native plant communities, and when a long time has elapsed since the introduction [44–47].

In this study, we explored if native dune species have evolved adaptative responses as a consequence of the interaction with the invasive South African species *Carpobrotus edulis* (L.) N.E.Br. (Aizoaceae) (hereafter *Carpobrotus*), introduced at least one century ago to NW Iberia. In a common garden experiment, we compared the reciprocal effects of competition between *Carpobrotus* plants from either European (invader) populations or from native African populations and the native Iberian species *Artemisia crithmifolia* L. (*A. campestris* L. ssp. *maritima* (DC.) Arcang.; hereafter *Artemisia*) and *Helichrysum picardii* Boiss. & Reuter (*Helichrysum serotinun* subsp. *picardii* (Boiss & Reuter) Galbany, L. Sáez & Benedí; hereafter *Helichrysum*). These are among the most representative endemic species from secondary or grey dunes, one of the main habitats invaded by *Carpobrotus* in NW Iberia. The sampled native plants were from populations that had either already been exposed to European *Carpobrotus* and have therefore had the opportunity to co-evolve with it (exposed populations) or they were from populations from the same region that had not been exposed to *Carpobrotus*. Considering the time elapsed since the introduction of *Carpobrotus* to the NW Iberian Peninsula, we hypothesized that it may have genetically changed to adapt to the new conditions. This same old invasion, along with the strong selection pressures exerted by an invader able to establish monodominant stands, may have resulted in natives' adaptions reducing the impact of the invader and easing the future development of a stable, biodiverse community.

## Results

**Fig. 1:**
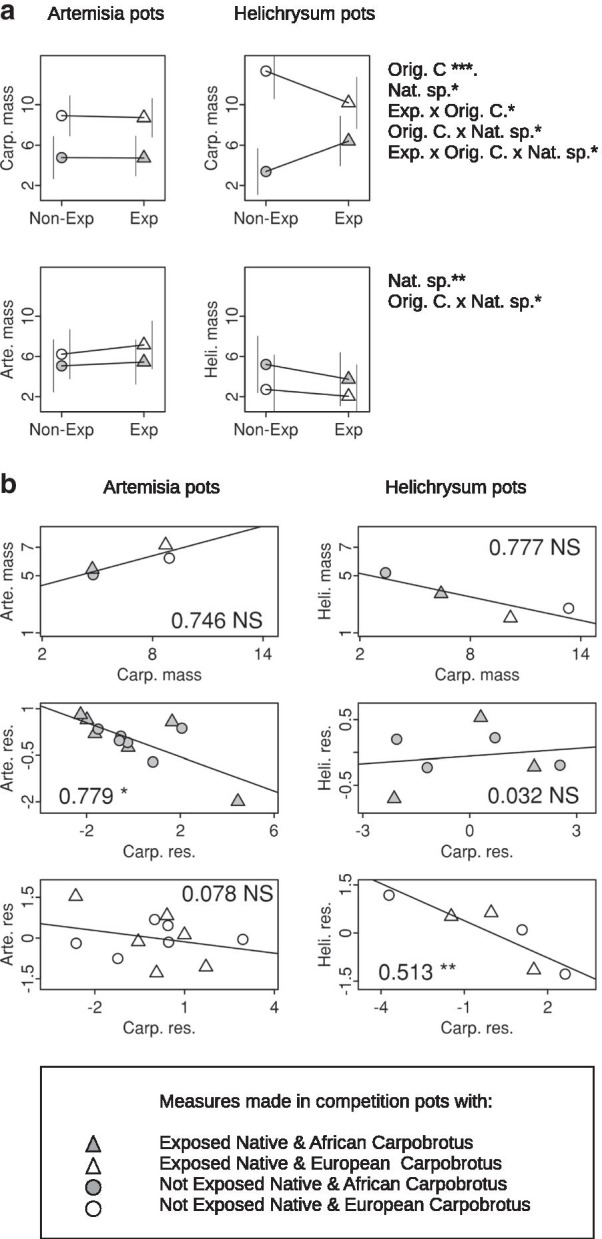
Least square means and residuals in the analysis of plant final dry mass in two plant pots (“Heli”, *Helichrysum*, and “Arte”, *Artemisia*). **a** Lsmeans for the *Carpobrotus* and native species’ masses. Vertical lines on the left-hand side and right-hand side of the graph show 95% vertical lines means asymptotic confidence intervals for pots containing African and European *Carpobrotus* respectively. Interval limits outside the comparison areas may lie outside the areas shown in the graphs. Right, summaries of the Table 1 analyses of *Carpobrotus* and natives’ masses. **b** Top, two-dimensional representation of the lsmeans in (**a**). All lsmeans correspond to untransformed data and are drawn to the same scale to ease comparisons. Middle and bottom, bidimensional representations of the residuals in the analysis of *Carpobrotus* and natives’ masses in pots containing African and European *Carpobrotus*, respectively. The r squared and the significance of the slope in a regression of natives’ on Carpobrotus’ residuals are shown on the graphs. *, *P* < 0.05; **, *P* < 0.01; ***, *P* < 0.001

**Fig. 2:**
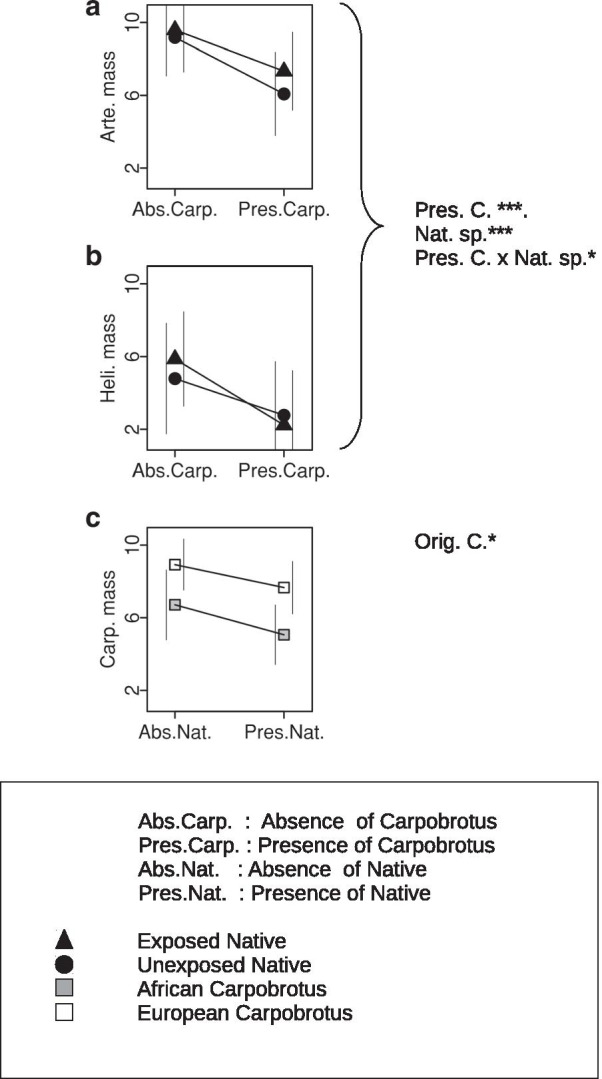
Least square means for whole plant dry mass in the comparisons of competition and single plant pots. Vertical lines on the left-hand side and right-hand side show 95% asymptotic confidence intervals for the lsmeans. Interval limits outside the comparison areas may lie outside of the areas shown in the graphs. Right, summaries of the Additional file 1: Table S1 analyses of *Carpobrotus* and native masses. *, *P* < 0.05; **, *P* < 0.01; ***, *P* < 0.001

No *Carpobrotus* or *Artemisia* plants died in the competition pots, whereas two exposed and two unexposed *Helichrysum* plants competing with the African *Carpobrotus* died, as did three exposed and three unexposed *Helichrysum* plants competing with the European *Carpobrotus.* We show in more detail the analyses corresponding to final whole plant dry mass, hereafter “growth” in Fig. 1 and Table 1 for the competition pots, and Fig. 2, Additional file 1: Table S1 and Additional file 2: Table S2 for the comparisons between competition and single plant pots. The corresponding results for shoot, root and whole plant dry mass were qualitatively similar to those for growth and are shown in Additional file 3: Table S3, Additional file 4: Table S4 and Additional file 5: Table S5.

**Table 1:** Analysis of the final dry massses of the native and *Carpobrotus* plants in the pots containing two plants

Effect	Final log mass of native species				Final mass of *Carpobrotus* plants			
	Advantage/*slope*	LRT *P*	Relative AIC weight	Selected model *P* value	Advantage/slope	LRT *P*	Relative AIC weight	Selected model *P* value
Exposure	Non-exposed	0.345	0.574	0.365	Unexposed	0.887	0.372	0.271
Origin of *Carpobrotus*	African	0.063	2.076	0.675	European	26 × 10^–6^	23 × 10^4^	1.000
Native species	*Artemisia*	0.004	24.115	0.960	*Helichrysum*	0.022	5.132	0.837
Initial Mass *Carpobrotus*	*0.097*	0.355	0.527	0.345	− 0.791	0.159	0.991	0.498
Initial Mass Native species	*0.024*	0.019	1.836	0.647	0.101	0.036	1.127	0.530
Exp. × Orig. C		0.396	0.528	0.345		0.018	6.070	0.858
Exp. × Nat. sp.		0.521	0.452	0.311		0.968	0.368	0.269
Ori C. × Nat. sp.		0.005	19.071	0.950		0.028	4.062	0.802
Exp. × Orig. C. × Nat. sp.		0.993	0.368	0.269		0.019	5.663	0.850

Columns show the levels of the main factors at advantage for final mass or the estimated slopes for the covariables, the Likelihood Ratio Tests probability for each model term, the AIC weight and the normalized probability that the model including that term is preferred. In both analyses, the residual degrees of freedom were 28 (only pots having data for the native and the *Carpobrotus* plant were analysed). The masses of native plants were logarithmically transformed to improve the normality and variance homogeneity of the model residuals. After this transformation, the masses of both native plants and *Carpobrotus* were normally distributed, as indicated by the Shapiro—Wilks normality test (*P* = 0.107 and *P* = 0.973) and Bartletts' homogeneity of variance test (*P* = 0.767 and *P* = 0.653)

### Exposure of native Iberian species to *Carpobrotus*

Exposure was not significant in either the native or *Carpobrotus* plants (Table 1). However, in the case of *Carpobrotus* growth*,* this was due to heterogeneity of results across *Carpobrotus* origin and native species. We found a significant Exposure × Origin of *Carpobrotus* effect of *Helichrysum* on *Carpobrotus*, but not of *Artemisia*. The African *Carpobrotus* grew more when competing with the previously exposed *Helichrysum*: analysis of data from pots containing *Helichrysum*/African *Carpobrotus* detected a significant (LRT *P* = 0.024) and positive effect of Exposure. The difference occurred in the opposite direction in the corresponding analysis for European *Carpobrotus* (Fig. 1, LRT *P* = 0.174). This variation resulted in a significant (LRT *P* = 0.021) Exposure × Origin of *Carpobrotus* interaction in an analysis restricted to pots shared by either *Carpobrotus* with *Helichrysum*. Thus, the exposed *Helichrysum* suppressed more the growth of the European *Carpobrotus* with which it had the opportunity to co-evolve. The corresponding analysis for *Artemisia* did not detect any such interaction (LRT *P* = 0.600), and the difference between native species for double interactions resulted in a significant triple Exposure x Origin of *Carpobrotus* × Native species interaction in the full model (i.e., in the joint analysis of all competition pots in the experiment; Fig. 1 and Table 1). Thus, the potentially coevolved *Helichrysum* had stronger effects on the growth of European *Carpobrotus* than the potentially coevolved *Artemisia*. No effects of Exposure or the corresponding interactions were observed in these comparisons of native plants in competition and single plant pots (Fig. 2 and Additional file 1: Table S1).

### Origin of *Carpobrotus*

The African *Carpobrotus* grew less than the European *Carpobrotus.* This was shown by the analysis of the competition pots (Fig. 1 and Table 1), the competition/single *Carpobrotus* plant pot comparisons (LRT *P* = 0.002, Additional file 2: Table S2 and Fig. 2) and also by the comparison of the African and European *Carpobrotus* grown in single plant pots (LRT *P* < 0.001). The Origin of *Carpobrotus* had no significant overall effect on the growth of the competing native plants, due to the heterogeneous growth of these plants. *Helichrysum* grew relatively more (on average for exposed and unexposed plants) in the presence of the African *Carpobrotus* than *Artemisia* (Fig. 1), as indicated by the significant Origin of *Carpobrotus* × Native species interaction (Table 1).

### Native species

The *Artemisia* plants grew more than the *Helichrysum* plants, as seen in the competition pot comparisons (Fig. 1 and Table 1) and confirmed by the comparisons between competition and single plant pots (Fig. 2 and Additional file 1: Table S1) and by the direct comparison of growth of each species in the single plant pots (LRT *P* < 0.001). The effect on *Carpobrotus* growth was also significant: the *Carpobrotus* plants competing with the larger *Artemisia* grew less than those competing with the smaller *Helichrysum* (Fig. 1 and Table 1).

### Competition

Both African and European *Carpobrotus* plants grew more when competing with *Helichrysum*, the smallest of the two native species studied (see Methods section), and therefore that expected to generate less competition for resources in the pots. This was consistent with competition limiting plant growth in this experiment. The bidimensional representation of the least square means from the analysis of the competition pots (Fig. 1) would support this view in the case of *Helichrysum*: the estimated correlation between mean growth of *Helichrysum* and *Carpobrotus* in the same pot was negative. This contrasted markedly with the positive sign of the corresponding estimate for *Artemisia*. As the power to detect four-point correlations is low, these two estimates were not significantly different from zero when tested separately. However, randomizing the allocation of pairs of standardized least square means to the two species resulted in only 144 replicates on 10,000 with larger than observed between-species differences in correlation, showing that the correlations between *Carpobrotus* and native plants were significantly (*P* = 0.014) different for the two species considered.

The residuals obtained after fitting the analytical model to the competition pots (Fig. 1) showed that some within-pot competition remained after correcting for the effect of Exposure, Origin of *Carpobrotus* and Native species. The correlation between the residuals of the *Carpobrotus* and native plant analyses were − 0.553 (*P* = 0.005, 22 d. f.) for *Artemisia* and − 0.558 (*P* = 0.048, 11 d. f.) for *Helichrysum*. The effect was not homogeneous in *Helichrysum* (Fig. 1). A separate estimate of this correlation for the competition pots with African *Carpobrotus* was positive (r = 0.176, *P* = 0.705, 5 d. f.), and another for the competition pots with European *Carpobrotus* was negative (r = − 0.883, *P* = 0.020, 4 d. f.), consistent with stronger competition between *Helichrysum* and the European *Carpobrotus*. A test randomizing the allocation of plant pair means to the two groups (of competition pots with one *Helichrysum* and one African or European *Carpobrotus*) detected only 211 of 10,000 replicates with more extreme differences in correlation. While this test was not planned a priori, the difference between the two correlations was remarkable. We found no evidence of such heterogeneity in the *Artemisia* pots, and the correlations were − 0.716 (*P* = 0.009, 10 d. f.) and − 0.278 (*P* = 0.381, 10 d. f.) in the pots with competing African and European *Carpobrotus*. The randomization test detected 1985 replicates of 10,000 with more extreme differences in correlation than observed between the two groups of *Artemisia* pots. There were no significant differences between the residual's correlations of exposed and unexposed weights in any species (*P* = 0.142 in a joint randomization test for both native species). The two native species’ contrasting correlations with *Carpobrotus,* both for lsmeans and residuals, suggest that their patterns, and possibly mechanisms of competition with *Carpobrotus* were different.

The Presence of *Carpobrotus* significantly depressed the growth of the native plants in the comparison of competition and single plant pots, whereas Presence of native plants did not significantly affect the growth of *Carpobrotus* (Fig. 2, Additional file 1: Table S1; this comparison did not use the unexposed natives; see Methods). In the same analysis, the significant interaction (LRT *P* = 0.044) between Presence of *Carpobrotus* and Native species again supports increased competition between *Helichrysum* and *Carpobrotus*.

**Fig. 3:**
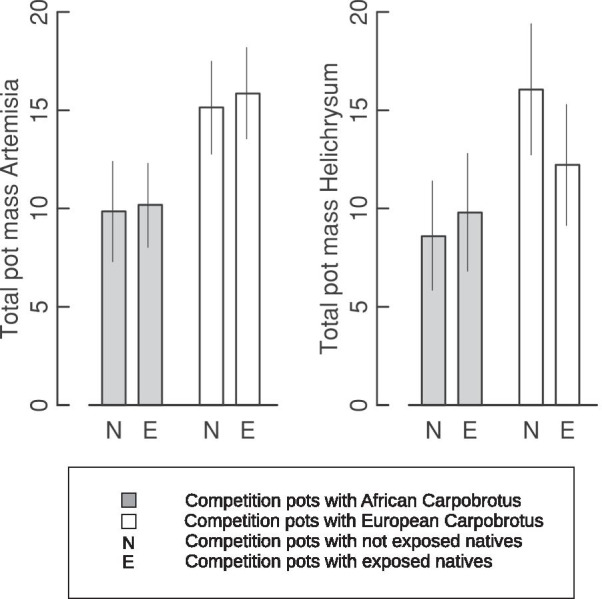
Least square means for total biomass (g) in the competition pots. The vertical lines show asymptotic 95% confidence intervals. All lsmeans correspond to untransformed data and are drawn to the same scale to ease comparisons

The total biomass in the competition pots (i.e., the sum of the weight of native plants and *Carpobrotus* plants) was greater in the pots containing European *Carpobrotus* (Origin of *Carpobrotus*, LRT based on the same model as used for the weights of native Iberian species and *Carpobrotus P* < 0.001; Fig. 3) but there were no differences between competition pots containing exposed and unexposed native plants.

## Discussion

The two native species showed different patterns of Exposure × Origin of *Carpobrotus* interaction, which was consistent with differences in adaptative responses to the invader. We had no evidence of such interaction, and therefore response, in *Artemisia*. Our experiment compared in a common greenhouse environment plants from two populations of each native species, one exposed and the other unexposed to *Carpobrotus*. It would be parsimonious to attribute any overall differences between these two populations to the random sampling of each species' interpopulation variation, and this variation could be the result of many processes, like local abiotic adaption or genetic drift, besides adaptation to *Carpobrotus* presence. However, we found no such overall differences -i.e., no significant main factor Exposure- either for the competitive effect of the native plants on *Carpobrotus*, or their response to *Carpobrotus*. The only effects of Exposure were specific for each origin of the competing *Carpobrotus*, African or invader. They were detected by the significant interaction Exposure × Origin of *Carpobrotus*, where the strong interaction for *Helychrysum* prevailed over the small or inexistent interaction for *Artemisia*, and the triple interaction Exposure × Origin of *Carpobrotus* × Native species, reflecting this heterogeneity between native species for the Exposure × Origin of *Carpobrotus* interaction. The specificity of these Exposure effects for the two origins of *Carpobrotus* makes the mere sampling of interpopulation variation a less parsimonious interpretation and clearly suggests adaptation of *Helichrysum* to the *Carpobrotus* invasion.

The relative increase in growth for the African *Carpobrotus* competing with the exposed *Helichrysum* suggests that this native’s response to the European *Carpobrotus* involves costs that reduce its performance when the invader is absent. In another potentially costly adaptation, fitness of populations of the native *Pilea pumila* exposed to the invader *Alliaria petiolata* was maximal in sites with high densities of the invader and minimal in the low-density sites [32], indicating that adaptation to interaction with invasive species may become counterproductive when the invaders are rare or absent.

The change observed in the exposed *Helichrysum* does not fit the predictions of the Atwater's model [48] of plant invasion and the observations by Fletcher et al. [49]. According to that model, of the two components of plant competitive ability defined by Miller and Werner [50], namely the ability of an individual plant to suppress competitors and the ability to tolerate them, competition among more than two individuals or species would favour the evolution of tolerance instead of suppression. This is because increased tolerance benefits only the species experiencing it, whereas increased suppression of some competitors would also benefit all no suppressed species in the competing assemblage. Consequently, the reduction in overall competition for the suppressor would be limited. The situation could be different in our experiment due to the asymmetry of the competition. *Carpobrotus* is a very successful invader and it could be difficult for native competitors to completely fill the void left by a suppression of its competitive effects. So, the *Helicrysum* plants could get a net benefit by trading *Carpobrotus* for other natives’ competition. However, no significant increase in growth was detected in the exposed *Helichrysum* competing with the European *Carpobrotus*.

The observed relative decrease in *Carpobrotus* growth by the exposed *Helichrysum* was modest and did not prevent the European *Carpobrotus* from becoming larger than the African plant. The larger mass was consistent with the trend of plants becoming taller and more vigorous when they grow in non-native environments [51] and with the evolution of increased competitive ability hypothesis [52]: plants will show trade-offs in resource allocation to growth, reproduction and defence [53, 54], so that release from the biological enemies in their native environment will enable the invaders to increase their investment in other traits. In that case, it would be remarkable that *Carpobrotus* had maintained such release for so many years since its introduction. It is possible that the absence of native plants phylogenetically close (see [55]) to *Carpobrotus* in the NW Iberian Peninsula made it difficult for local phytophagous and pathogenic species to extend the range of exploited plants to encompass the newcomer.

**Fig. 4:**
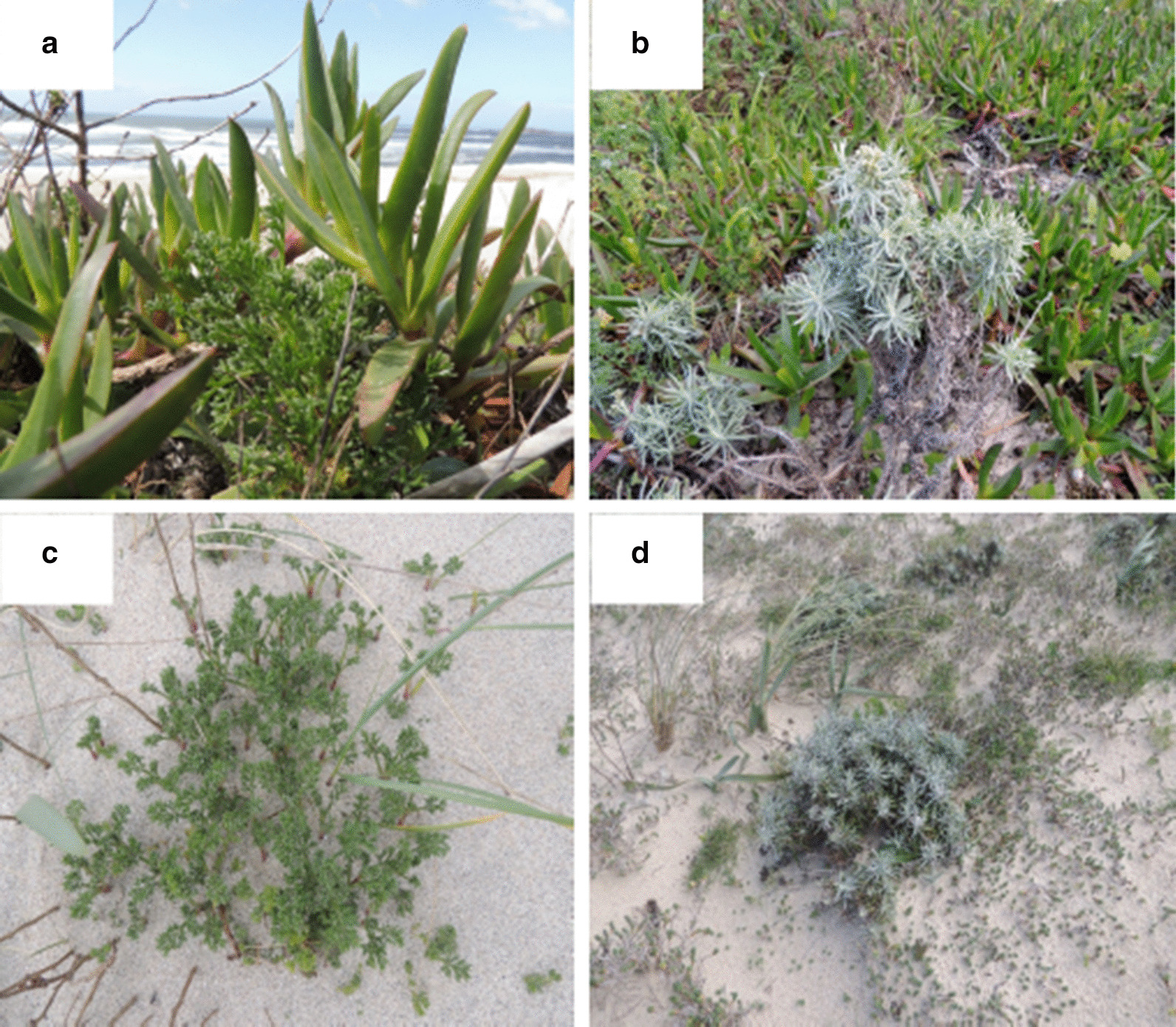
*Carpobrotus* plants and native plants in the locations sampled. *Artemisia crithmifolia* (**a**) and *Helichrysum picardii* (**b**) plants exposed to *Carpobrotus* in Praia de Moledo; **c** and **d** *A. crithmifolia and H. picardii* from the populations unexposed to *Carpobrotus* in Praia do Trece and Praia das Furnas, respectively

The comparison of competition and single plant pots revealed high levels of competition in the pots containing two plants for both native species. Despite this competition, the greater dry mass of the European *Carpobrotus* was not obtained completely at the expense of the native plants, as the total biomass in the pots containing one native and one European *Carpobrotus* plant was higher than in those containing one native and one African *Carpobrotus* plant. This raises the possibility that the primary productivity of plant communities may have increased since the introduction of *Carpobrotus*. It must be noted however that conditions in the greenhouse cannot perfectly reproduce those in the field. For example, we tried to maintain all pots optimally watered thorough the experiment, thus excluding root competition for water, which could play some role in the interspecific competition in the field. Similarly, the regular arrangement of plants in the pots could not fully represent the irregular plant distribution and density observed in the dunes. However, as seen in Fig. 4, distances between plants in the field may be as short as in our experimental pots (uncharacteristically isolated plants were chosen for the pictures of non-exposed plants to improve visibility).

Two kinds of competitive interactions would have occurred in the pots. First, exploitation or scramble competition, where mineral soil resource availability to competitors is affected through resource depletion, and second, interference or contest competition, where interaction occurs through the production and release into the soil of chemicals that are toxic to other species or inhibit access of other roots to resources (allelopathy). Some previous studies have demonstrated that both mechanisms play a key role driving the competition between *Carpobrotus* and native species in the field [56]. The correlation between the lsmeans of native and *Carpobrotus* plants in the same competition pot provided some evidence for interference. Because *Artemisia* is the largest of the two natives and the one suppressing *Carpobrotus* growth the most, it would be expected to be involved in stronger resource competition and more negative lsmeans correlations with *Carpobrotus*: pots with large *Artemisia* plants would be expected to sustain smaller *Carpobrotus*, and vice versa. But it was the reverse. The correlation was significantly less negative than that for *Helichrysum*. This reduced dependency of competition on plant size in *Artemisia* would be consistent with this species’ biology. While there is some evidence of allelopathic properties in the genus *Helichrysum* [57], direct comparison of allelopathic activities in plants of the *Helicrysum* and *Artemisia* genera [58] revealed a clear advantage in the activity of the latter. Allelopathy could thus explain a depression in *Carpobrotus* growth that is not dependent on the variation in size of *Artemisia*, as there could be differences in the regulation of plant growth and allelopathic activity. In fact, trade-offs between growth and production of allelopathic compounds have been found, at least in seaweeds [59].

Some evidence of competition remained after adjusting for the main effects Exposure and Origin of *Carpobrotus* and their interactions in the analytical model, as shown by the mainly negative correlations between the residuals for *Carpobrotus* and native species from the model adjusted to analyse their growth. These correlations were consistently negative for the *Artemisia* data, thus indicating that, although not dependent on the Exposure or Origin of *Carpobrotus* considered in that model, competition for limited resources in the pots occurred between *Artemisia* and *Carpobrotus*. In any case, the observation that the presence of *Carpobrotus* generally depressed the growth of *Helichrysum* more than that of *Artemisia* in the comparison of competition and single plant pots suggested more intense competition for resources. This could have resulted in stronger selection pressures on *Helichrysum* populations to adapt to the presence of *Carpobrotus*. Differences in the competitive impact of *Carpobrotus* across native species had already been observed in field studies of invaded areas [60, 61].

The difference in the response of the native species to the introduction of *Carpobrotus* could be related also to the genetic structure of the populations of these species*. Artemisia* displays very limited genetic variation, both between and within populations, in the study region, probably due to its ability to disperse over long-distances at high rates, and to initiate new populations from very small propagules, in a series of founder events [62]. The low variation will limit the potential of exposed subpopulations to adapt to competition from the invasive *Carpobrotus*. By contrast, *Helichrysum italicum* has been shown to maintain considerable genetic differences between subpopulations at distances of only tens of kilometres in Sardinia, probably due to limited mobility of pollinating insects [63], and considerable variation within populations, at least in the Western Mediterranean [64]. Similar partial isolation could facilitate the local evolution of resistance in areas of the NW Iberian Peninsula invaded by *Carpobrotus*. Interestingly, *Helichrysum italicumm* subsp. *picardii* was the second most abundant native species in a study of 8 sites invaded by *Carpobrotus* in the sand dune systems of the western coast of Portugal (with an average cover of 6.0%, compared with 6.6% for *Corema album*, 13.9% for *Carpobrotus edulis* and the only *Artemisia* species mentioned (*Artemisia campestris* ssp. *maritima*) was in eighth position, with 1.7% [65]). A large population size favours the maintenance of genetic variation, which would also facilitate the evolution of resistance to *Carpobrotus*. However, it is not clear whether the large populations of *Helichrysum* in the *Carpobrotus*-invaded sites are the cause or the consequence of that evolution, as we are not aware of any comparison of *Helichrysum* abundance in invaded and non-invaded sites.

These evolutionary considerations may be useful additions to the list of criteria for assessing the vulnerability of native species and ecosystems to biological invasions, on which to base the assignment of priorities for surveillance and protection interventions [66]. These assessments tend to be based on ecological features (e.g. [67, 68], but our study suggests that vulnerability to biological invasions may also depend on the genetic structure of populations, the amount of genetic diversity and the gene flow patterns. The same factors could also be important for designing management plans for invasive species [69].

## Conclusions

In conclusion, we found that native species can respond in order to reduce the ecological impact of invasive species, which would facilitate the integration of the latter into the invaded community. This result is consistent with previous studies of ancient introductions of mussel macroparasites (~ 70 years [70]), herbaceous plants (~ 150 years [71]) and trees (~ 170 years [14]). However, these changes may only occur in some native species, possibly depending not only on ecological, but also on evolutionary aspects such as population size and the amounts of genetic variance and gene flow. We propose that consideration of these aspects may be important in analysing the conservation impact of biological invasions. The heterogeneity in native plant responses to invasion might help to explain why *Carpobrotus* is still having a strong impact across its area of distribution in NW Iberia.

## Methods

### Introduction of *Carpobrotus edulis*

*Carpobrotus edulis* is a succulent perennial plant that has been introduced from its native range in South Africa [72] across all Mediterranean climate regions, including California, Australia and the Mediterranean basin [73]. In Europe, this species has been grown for ornamental purposes since the beginning of the seventeenth century [74] and records of its presence in NW Iberia date back to the eighteenth century [73]. Due to its ability to rapidly spread forming deep, dense mats, the species has been used to stabilize sand dunes and prevent soil erosion in this area since the early twentieth century and nowadays naturalized populations of *C. edulis* can be found elsewhere in coastal habitats [73], where it may have been co-evolving with native plants for more than 100 years. Its facultative C3-CAM physiology [75], high morphological and ecophysiological plasticity [76–78], flexible mating system [79], and an intense vegetative clonality [20, 73, 80], enable the plant to tolerate a wide range of ecological conditions. These characteristics along with the high rates of seed dispersal [81], are also important features explaining the effective colonization of dune habitats, where plants compete for space, light, water and nutrients [82] in such a way that *C. edulis* can reduce the growth, survival, and reproduction of some native species [73] and references therein]. Consequently, the release of *Capobrotus* in natural environments and protected areas is prohibited in several countries (e.g., Spain, Portugal, United Kingdom, Ireland, and Italy), although this taxon is not included in the Regulation (EU) no. 1143/2014 [83]. In California, the plant poses a threat to several rare and endangered plant species and it is listed as CalEPPC List A-1 and as CDFA-NL (http://www.cal-ipc.org/); on the contrary it is not declared or considered noxious by any state government authorities in Australia [73].

### Plant sampling

We collected *Carpobrotus* plants from sand dune populations in their South African native range (Hawston beach, 34° 23′S, 19° 07′W, Western Cape, South Africa) in mid-January 2015 and in the invaded range (Praia de Moledo, 41º 51′N, 8º 51′W, Caminha, Portugal) in mid-April 2015. The African specimens of *Carpobrotus* were used as an experimental control as they share origins with the invasive European *Carpobrotus*, but not their recent adaptive story. The *Artemisia crithmifolia* and *Helichrysum picardii* plants exposed to *Carpobrotus* were collected from Caminha (Portugal) at the same time as the *Carpobrotus* plants.

The two native species selected in the experiment differ in several respects relevant for their evolutionary responses to biological invasion. *Artemisia* is a larger plant (see the two species’ descriptions in Castroviejo et al. [84]) with more allelopathic activity [58] and lower genetic variation [62, 63] and ground cover in the sampled sand dunes [65]. *Artemisia,* but not *Helichrysum*, has rhizomatous structures allowing to optimize belowground resource uptake and storage, which could increase its competitive ability. The *Artemisia* and *Helichrysum* plants from populations unexposed to *Carpobrotus* were collected in mid-March 2016, in Camariñas (Praia do Trece, [43° 11′N, 9º 10′W], Galicia) and Porto do Son (Praia das Furnas [42° 38′N, 9º 02′W], Galicia), respectively (Fig. 4). The population of *Artemisia* in Porto do Son and the population of *Helichrysum* in Camariñas were too small to take a representative sample of both species from one site. Thereby, unexposed *Artemisia* and *Helichrysum* plants were collected in Camariñas and Porto do Son, respectively. The distance between these populations is about 76 km and the environmental conditions are quite similar. The monthly mean temperatures registered at the meteorological station of Camariñas ranged from 11 ºC (March 2016) to 18.5 ºC (August 2016) and monthly mean rainfall ranged from 397.1 L/m^2^ (January 2016) to 1.3 L/m^2^ (July 2016). The monthly mean temperatures registered at the meteorological station of Ribeira, a place very near to Porto do Son, ranged from 10.9 ºC (February 2016) to 21.5 ºC (July 2016) and monthly mean rainfall ranged from 265.7 L/m^2^ (January 2016) to 4.8 L/m^2^ (July 2016) (www.meteogalicia.es). No direct measures of the extent of *Carpobrotus* plant cover are available for the exposed sampling sites, but visual estimates based on pictures taken during the sampling were of about 60–70% in both sites.

We sampled intensively the native and invasive *Carpobrotus* populations. To have a more comprehensive representation of the genetic variability of the species in each area, we selected 36 separated clumps per area. The minimum separation between sampled clumps was of 25 m. *Carpobrotus* forms compact clumps [72] and it is reasonable to assume that each separated clump represents a different genotype. Thus, we would have collected a total of 72 genotypes. From these, we randomly selected the genotypes used in our experiment. Our sampling protocol has been described in detail in Roiloa et al. [20]. Likewise, the populations of the two native species were extensively sampled in order to gather the greatest genetic diversity inside each population.

The plant taxa nomenclature follows standard Iberian floras [84]. The native species were collected following current Spanish regulations. No specific permissions were required. The invasive *C. edulis* was collected from natural populations and propagated under the permission from the Spanish Ministry of Agriculture, Food, and the Environment, and complied with the Convention on the Trade in Endangered Species of Wild Fauna and Flora.

We found no historical records (including records about past eradication campaigns, which mainly began in the twenty-first century in NW Iberia; [73]) of the presence of *Carpobrotus* in our unexposed locations of Camariñas and Porto do Son. In any case, a previous, undocumented presence of *Carpobrotus* in the locations -both currently free of *Carpobrotus *- would imply that the species had become extinct, which is unlikely given its invasive nature. Collected plants were washed and maintained in a climate-controlled greenhouse at the University of Santiago de Compostela until the start of the experiment in April 2016.

### Experimental design

The experiment was carried out in a greenhouse at the University of Santiago de Compostela (Galicia, Spain). We used 5L plastic pots filled with a growing substrate similar to that in natural conditions, i.e., a 1:1 mixture of potting compost and dune sand. The environmental conditions were identical for all species, grown under a natural day/night light cycle between April 2016 and April 2017. Monthly global irradiance ranged from 15.3 MJ m^−2^ day^−1^ (April 2016) to 21.3 MJ m^−2^ day^−1^ (April 2017), although photosynthetically active radiation was reduced by about 12% inside the greenhouse with respect to full sunlight outdoors (measured with a LI-190SA Quantum Sensor, LI-COR, Lincoln, Nebraska, USA). The temperature inside the greenhouse ranged from 15 ℃ to 22 ℃. The plants were watered according to their requirements (once or twice per week) in order to prevent hydric stress. Additionally, to avoid confounding effects of pot position within the greenhouse, these positions were randomized monthly.

**Fig. 5:**
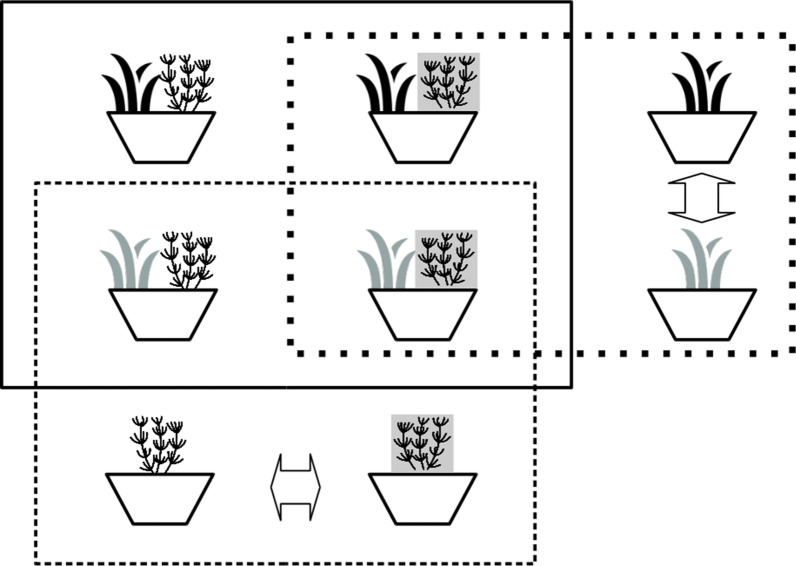
Experimental set-up. The experimental units were pots containing African or European (black and grey) *Carpobrotus* and native Iberian plants previously exposed or not previously exposed (grey and white background) to European *Carpobrotus*. There were two sets of pots as shown, one for each native species. The plot lines and arrows mark the pots used in the data analyses: continuous line, comparisons of pots containing two plants; fine dashed lines, comparisons between native Iberian plants in pots containing one and two plants; thick dashed lines, comparisons between *Carpobrotus* plants in pots containing one and two plants; block arrows, comparisons between plants in pots containing one plant. Icons from [86, 87]

The basic units in our experiment were competition pots containing one native plant and one *Carpobrotus* genet. Each genet, obtained from different donor plants*,* was composed by the three-vegetative most apical ramets (i.e., modules sensu Harper [85]), to guarantee that all of the material was at the same development stage. The plant pairs in these competition pots were arranged in a factorial design (Fig. 5) considering the effects of prior exposure of native plants to *Carpobrotus* (Exposure: exposed/unexposed), origin of *Carpobrotus* plants (African/European), native Iberian plant species (*Artemisia*/*Helichrysum*) and their interactions on the competition between the two plants (six replicate pots per combination of factors: 6 × 2 × 2 × 2 = 48 pots; 96 plants). The two-plant competition pots were complemented with pots containing single plants (six pots for African *Carpobrotus*, six for European *Carpobrotus*, and six for each Exposure × Native species combination: 36 pots and plants). Comparisons between single plant and competition pots made possible to confirm the existence, and measure the intensity, of competition experienced by *Carpobrotus* and native plants in the competition pots.

### Measured variables

Before the start of the experiment, the initial fresh weight of each *Carpobrotus*, *Artemisia* and *Helichrysum* plant was measured to the nearest 0.0001 g (Mettler AJ100, Mettler-Toledo, Greifensee, Switzerland). The plants were grown for twelve months under the experimental conditions and were then harvested, washed, cleaned and dried at 60 °C to constant weight. Each plant was separated into shoots (including leaves and stolons in the case of *Carpobrotus*) and roots, and the final dry weights of each fraction (total, above ground and root, dry mass) were recorded.

### Data analysis

All statistical analyses were based on linear models. The data sets were unbalanced, as some plants did not survive up to the end of the experiment, and we used the R [88] package stats’ “glm()”, “drop1()” and “emmeans()” functions to carry out likelihood ratio tests (LRT) and calculate least square means (hereafter “lsmeans”) for the mass data. We complemented the LRTs with more intuitive, Akaike weight-based calculations of the probability of one model favouring the other [89]. The difference in AIC between model i and the best model, i.e., that with minimum AIC, is

∆_*i*_*(AIC)* = *AIC*_*i*_* – minAIC*

and the weight of model i:

$$w_{i} \left( {{\text{AIC}}} \right) = \frac{{{\text{exp}}\left\{ {\frac{ - 1}{2}\Delta_{i} \left( {{\text{AIC}}} \right)} \right\}}}{{\mathop \sum \nolimits_{{{\text{k = }}1}}^{K} {\text{exp}}\left\{ {\frac{ - 1}{2}\Delta_{k} \left( {{\text{AIC}}} \right)} \right\}}}$$

where K is the number of models considered. The normalized probability that model 1 is preferred (i.e., it is better in terms of Kullback–Leibler discrepancy; see [90]) over model 2 is

$$w_{1} \left( {AIC} \right) \, / \, \left( {w_{1} \left( {AIC} \right) \, + \, w_{2} \left( {AIC} \right)} \right)$$

Because the numbers of parameters in the statistical models considered were large relative to sample sizes, we replaced AIC with its small sample version AIC_c_ [90] in all calculations shown.

Both the analyses of the measures from the native plants and the *Carpobrotus* plants in the competition pots considered the effects of Exposure, Origin of *Carpobrotus*, Native species and their interactions. This was because, in a competition situation, the characteristics of one plant may affect the plant competing with it. For example, African and European *Carpobrotus* could have different effects on the native plant in the same pot. For the same reason, both analyses considered as covariables the initial fresh mass of the native and the *Carpobrotus* plants.

The native Iberian species growing with South African *Carpobrotus* were not considered in the comparisons between the native plants in competition and single plant pots, because this would have yielded heterogeneous and difficult-to-interpret levels for the origin of *Carpobrotus* factor (European, African and none). The effects considered in these analyses were Exposure to *Carpobrotus,* Native species and the Presence (presence/absence) of *Carpobrotus* in the pot. Only the initial fresh mass of the native plants was used as a covariable here, as only half of the pots (those containing two plants) contained a *Carpobrotus* plant for which an initial weight was available. Similarly, the *Carpobrotus* plants growing with non-exposed native plants were not considered in the comparisons between *Carpobrotus* in competition and single plant pots, to prevent heterogeneous and difficult-to-interpret levels for the Exposure factor (exposed, unexposed and no native plant). Only the initial fresh mass of *Carpobrotus* was used as a covariable in these analyses, along with Origin of *Carpobrotus* and Presence of native Iberian plants.

## Supplementary Information

**Additional file 1: Table S1. **Analysis of the final dry masses of the native plants in the comparison of pots containing one plant and two plants. Columns show the levels of the main factors in advantage for final mass or the estimated slopes for the covariables, and Likelihood Ratio Tests probability for each model term, AIC weight and the normalized probability that the model including that term will be selected. Number of residual degrees of freedom =27.

**Additional file 2: Table S2. **Analysis of the final dry masses of the *Carpobrotus* plants in the comparisons of pots with one plant and two plants. Columns show the levels of the main factors in advantage for the final mass or the estimated slopes for the covariable, and Likelihood Ratio Tests probability for each model term, AIC weight and the normalized probability that the model including that term will be selected. Number of residual degrees of freedom =16.

**Additional file 3: Table S3. **Likelihood Ratio test probabilities for mass-related variables in the comparisons of pots containing two plants.

**Additional file 4: Table S4. **Likelihood Ratio tests probabilities for mass-related variables for native Iberian species in the comparisons of pots containing one and two plants.

**Additional file 5: Table S5. **Likelihood Ratio tests probabilities for mass-related *Carpobrotus* variables in the comparisons of pots containing one and two *Carpobrotus* plants.

**Additional file 6:** The full information on sampling locations, experimental treatments, initial and final fresh masses and final root and total dry masses for all native and *Carpobrotus* plants in the experiment.

## Data Availability

The dataset supporting the conclusions of this article is included within the article (and its Additional file 6).
