# Supplementary material for: A test of native plant adaptation more than one century after introduction of the invasive Carpobrotus edulis to the NW Iberian Peninsula
Source: BMC Ecol Evol. 2021 Apr 28;21:69. doi: 10.1186/s12862-021-01785-x (PMC8080363; doi:10.1186/s12862-021-01785-x)
Supplement: Supplementary file 1 — Additional file 1: Table S1. Analysis of the final dry masses of the native plants in the comparison of pots containing one plant and two plants. Columns show the levels of the main factors in advantage for final mass or the estimated slopes for the covariables, and Likelihood Ratio Tests probability for each model term, AIC weight and the normalized probability that the model including that term will be selected. Number of residual degrees of freedom =27. [file 12862_2021_1785_MOESM1_ESM.docx]

**Additional file 1. Table S1.** Analysis of the final dry masses of the native plants in the comparison of pots containing one plant and two plants.

Columns show the levels of the main factors in advantage for final mass or the estimated slopes for the covariables, and Likelihood Ratio Tests probability for each model term, AIC weight and the normalized probability that the model including that term will be selected. Number of residual degrees of freedom =27.

| **Effect** |  |  |  |  |
| --- | --- | --- | --- | --- |
|  | **Advantage / *slope*** | **LRT P** | **Relative AIC weight** | **Selected model P** value |
| Exposure | Exposed | 0.771 | 0.384 | 0.277 |
| Presence of *Carpobrotus* | Not present | 68 e-6 | 1013.6 | 0.999 |
| Native species | *Artemisia* | 26 e-6 | 2578.9 | 0.999 |
| Initial Mass of Native | *-0.04* | 0.007 | 14.605 | 0.936 |
| Exp. x Presence |  | 0.634 | 0.412 | 0.292 |
| Exp. x Nat. sp. |  | 0.819 | 0.378 | 0.274 |
| Presence x Nat. sp. |  | 0.044 | 2.807 | 0.737 |
| Exp. x Presence x Nat. sp. |  | 0.340 | 0.580 | 0.367 |
