# Supplementary material for: A test of native plant adaptation more than one century after introduction of the invasive Carpobrotus edulis to the NW Iberian Peninsula
Source: BMC Ecol Evol. 2021 Apr 28;21:69. doi: 10.1186/s12862-021-01785-x (PMC8080363; doi:10.1186/s12862-021-01785-x)
Supplement: Supplementary file 2 — Additional file 2: Table S2. Analysis of the final dry masses of the Carpobrotus plants in the comparisons of pots with one plant and two plants. Columns show the levels of the main factors in advantage for the final mass or the estimated slopes for the covariable, and Likelihood Ratio Tests probability for each model term, AIC weight and the normalized probability that the model including that term will be selected. Number of residual degrees of freedom =16. [file 12862_2021_1785_MOESM2_ESM.docx]

**Additional file 2. Table S2.** Analysis of the final dry masses of the *Carpobrotus* plants in the comparisons of pots with one plant and two plants.

Columns show the levels of the main factors in advantage for the final mass or the estimated slopes for the covariable, and Likelihood Ratio Tests probability for each model term, AIC weight and the normalized probability that the model including that term will be selected. Number of residual degrees of freedom =16.

| Effect | Advantage / slope | LRT P | Relative AIC weight | Model preferred with P |
| --- | --- | --- | --- | --- |
| Presence of native Iberian species | No presence | 0.306 | 0.622 | 0.383 |
| Origin of *Carpobrotus* | European | 0.002 | 35.229 | 0.972 |
| Initial Mass *Carpobrotus* | *0.243* | 0.078 | 1.734 | 0.634 |
| Presence x Orig. C. |  | 0.304 | 0.623 | 0.384 |
