# Supplementary material for: A test of native plant adaptation more than one century after introduction of the invasive Carpobrotus edulis to the NW Iberian Peninsula
Source: BMC Ecol Evol. 2021 Apr 28;21:69. doi: 10.1186/s12862-021-01785-x (PMC8080363; doi:10.1186/s12862-021-01785-x)
Supplement: Supplementary file 3 — Additional file 3: Table S3. Likelihood Ratio test probabilities for mass-related variables in the comparisons of pots containing two plants. [file 12862_2021_1785_MOESM3_ESM.docx]

**Additional file 3. Table S3.** Likelihood Ratio test probabilities for mass-related variables in the comparisons of pots containing two plants.

| Effect | Measures of native species mass | | | | Measures of *Carpobrotus* mass | | | | |
| --- | --- | --- | --- | --- | --- | --- | --- | --- | --- |
|  | Total fresh mass^L^ | Dry root mass^L^ | Total dry mass^L^ | Root:mass proportion^L^ | Total fresh mass^L^ | Dry root mass^L^ | Dry above ground mass^L^ | Total dry mass | Root: mass proportion |
| Exposure | 0.319 | 0.670 | 0.345 | 0.513 | 0.008 | 0.963 | 0.308 | 0.887 | 0.437 |
| Origin of *Carpobrotus* | 0.609 | 0.400 | 0.063 | 0.282 | 0.016 | 0.006 | 94 e-6 | 26 e-7 | 0.343 |
| Native species | 0.057 | 68 e-5 | 0.004 | 0.666 | 0.794 | 0.327 | 0.373 | 0.022 | 0.342 |
| Initial Mass *Carpobrotus* | 0.987 | 0.397 | 0.355 | 0.010 | 0.163 | 0.306 | 0.220 | 0.159 | 0.698 |
| Initial Mass Native species | 0.170 | 0.073 | 0.019 | 0.981 | 0.690 | 0.149 | 0.052 | 0.036 | 0.049 |
| Exp. x Orig. C. | 0.506 | 0.757 | 0.396 | 0.751 | 0.236 | 0.951 | 0.026 | 0.018 | 0.276 |
| Exp. x Nat. sp. | 0.576 | 0.193 | 0.521 | 0.196 | 0.026 | 0.374 | 0.272 | 0.969 | 0.363 |
| Orig. C. x Nat. sp. | 0.024 | 0.006 | 0.005 | 0.139 | 0.797 | 0.362 | 0.300 | 0.028 | 0.780 |
| Exp. Orig. C. x Nat. sp. | 0.734 | 0.478 | 0.993 | 0.203 | 0.104 | 0.216 | 0.090 | 0.019 | 0.205 |

^L^: Logarithmically transformed.
