# Supplementary material for: A test of native plant adaptation more than one century after introduction of the invasive Carpobrotus edulis to the NW Iberian Peninsula
Source: BMC Ecol Evol. 2021 Apr 28;21:69. doi: 10.1186/s12862-021-01785-x (PMC8080363; doi:10.1186/s12862-021-01785-x)
Supplement: Supplementary file 4 — Additional file 4: Table S4. Likelihood Ratio tests probabilities for mass-related variables for native Iberian species in the comparisons of pots containing one and two plants. [file 12862_2021_1785_MOESM4_ESM.docx]

**Additional file 4. Table S4.** Likelihood Ratio tests probabilities for mass-related variables for native Iberian species in the comparisons of pots containing one and two plants.

| Effect | Dry root mass^L^ | Dry above ground mass^L^ | Total dry mass^L^ | Root dry mass proportion^L^ |
| --- | --- | --- | --- | --- |
| Exposure | 0.899 | 0.859 | 0.771 | 0.967 |
| Presence of *Carpobrotus* | 0.008 | 0.002 | 68 e-6 | 0.424 |
| Species | 59 e-5 | 31 e-6 | 26 e-6 | 0.001 |
| Initial Mass Native | 0.106 | 0.005 | 0.007 | 0.026 |
| Exp. x PresC | 0.574 | 0.819 | 0.634 | 0.217 |
| Exp. x Nat. sp. | 0.548 | 0.743 | 0.819 | 0.693 |
| PresC x Nat. sp. | 0.202 | 0.074 | 0.044 | 0.333 |
| Exp. x PresC x Nat. sp. | 0.622 | 0.730 | 0.340 | 0.494 |

^L^: Logarithmically transformed.
