# Supplementary material for: A test of native plant adaptation more than one century after introduction of the invasive Carpobrotus edulis to the NW Iberian Peninsula
Source: BMC Ecol Evol. 2021 Apr 28;21:69. doi: 10.1186/s12862-021-01785-x (PMC8080363; doi:10.1186/s12862-021-01785-x)
Supplement: Supplementary file 5 — Additional file 5: Table S5. Likelihood Ratio tests probabilities for mass-related Carpobrotus variables in the comparisons of pots containing one and two Carpobrotus plants. [file 12862_2021_1785_MOESM5_ESM.docx]

**Additional file 5. Table S5.** Likelihood Ratio tests probabilities for mass-related *Carpobrotus* variables in the comparisons of pots containing one sand two *Carpobrotus*-plants.

| Effect | Dry root mass^L^ | Dry above ground mass^L^ | Total dry mass^L^ | Root dry mass proportion^L^ |
| --- | --- | --- | --- | --- |
| Presence of Native Iberian species | 0.503 | 0.219 | 0.306 | 0.109 |
| Origin of *Carpobrotus* | 0.091 | 0.002 | 0.002 | 0.314 |
| Initial mass of *Carpobrotus* | 0.100 | 0.083 | 0.078 | 0.712 |
| PresN x Orig. C. | 0.261 | 0.323 | 0.304 | 0.744 |

^L^: Logarithmically transformed.
